# Toward Endotype-Based Bronchopulmonary Dysplasia: Evolving Definitions and the Role of Translational Models

**DOI:** 10.3390/biomedicines14102145

**Published:** 2026-09-23

**Authors:** Marianela Rodriguez-Rey, Valeria Valez, Mauricio T. Caballero, Damián Alvarez-Paggi

**Affiliations:** 1 Departamento de Neonatología, Hospital de Clínicas, Facultad de Medicina, Universidad de la República, Montevideo CP 11600, Uruguay; 2 Centro de Investigaciones Biomédicas (CEINBIO), Universidad de la República, Montevideo CP 11600, Uruguay; 3 Departamento de Biología Odontológica, Subunidad Bioquímica y Biofísica Facultad de Odontología, Universidad de la República, Montevideo CP 11600, Uruguay; 4 Centro Infant de Medicina Traslacional (CIMeT), Escuela de Bio y Nanotecnologías (EByN), Universidad Nacional de San Martín (UNSAM), General San Martín CP 1650, Provincia de Buenos Aires, Argentina; 5 Consejo Nacional de Investigaciones Científicas y Técnicas (CONICET), Buenos Aires ZIP 1425, Argentina; 6 Centro de Rediseño e Ingeniería de Proteínas (CRIP), Escuela de Bio y Nanotecnologías (EByN), Universidad Nacional de San Martín (UNSAM), General San Martín CP 1650, Provincia de Buenos Aires, Argentina

**Keywords:** bronchopulmonary dysplasia, hyperoxia, premature infant, hyperoxia-induced lung injury, animal models, translational research

## Abstract

Bronchopulmonary dysplasia (BPD) remains one of the most frequent and clinically relevant chronic respiratory complications of extreme prematurity. Although successive definitions have improved disease classification, most are still based on oxygen requirement and respiratory support assessed at 36 weeks postmenstrual age (PMA), an approach that fails to capture the biological heterogeneity, dynamic clinical trajectories and long-term consequences of the disease. This narrative review analyzes the evolution of BPD definitions, from early time-based criteria to contemporary classifications based on severity and respiratory support, highlighting their strengths and limitations for predicting outcomes and translational research. We argue for the need to move beyond BPD as a single clinical entity toward a framework based on phenotypes and endotypes, including the parenchymal, airway, vascular, inflammatory, and mechanical components of the disease. Although non-animal alternatives are rapidly evolving, animal models remain the primary tools for analyzing specific developmental windows, injurious exposures, and molecular pathways involved in impaired alveolarization, abnormal vascular growth, inflammation, and ventilator-induced lung injury. We propose an endotype-based approach to guide the selection of preclinical models, supported by standardized injury protocols, longitudinal multimodal measurements, and the integration of emerging technologies such as organoids, lung-on-a-chip platforms, multi-omics, and predictive modeling. Redefining BPD through a translational and mechanism-based framework may improve risk stratification and support precision neonatal therapies.

## 1. Introduction

Bronchopulmonary dysplasia (BPD) remains a major challenge in neonatology. As the most common chronic respiratory complication of prematurity, it reflects a complex interplay between immature lung development and exposure to multiple prenatal and postnatal insults [1]. Advances in neonatal care, including antenatal corticosteroids, early surfactant therapy, and modern respiratory support strategies, have markedly improved the survival among preterm infants, particularly those with lower gestational age [2]. However, this progress has not been accompanied by a proportional reduction in the incidence of BPD. The incidence of BPD remains highly variable across centers and countries, with reported rates ranging from 10% to 89% [3,4,5]. The wide reported range of BPD incidence reflects differences in gestational age and birthweight distributions, diagnostic criteria, center-specific care practices, and survival to 36 weeks postmenstrual age (PMA), as competing early mortality may exclude the most vulnerable infants from incidence estimates [1,3,6]. This susceptibility partly reflects the fact that extremely preterm infants, while benefiting from life-sustaining interventions such as oxygen therapy and invasive mechanical ventilation, are still vulnerable to ventilator-induced lung injury, oxygen toxicity and inflammation [1]. In addition, common conditions of prematurity, including patent ductus arteriosus, can result in increased pulmonary blood flow, interstitial edema and inflammation, further amplifying injury in the developing lung [7]. BPD is associated with significant short and long-term health complications, including the need for prolonged respiratory support, pulmonary hypertension, recurrent respiratory disease, and adverse neurodevelopmental outcomes [8,9,10,11]. Together, the biological and clinical heterogeneity of BPD and the limitations of current definitions make it difficult to delineate distinct phenotypes and to establish more effective preventive and therapeutic strategies. This ultimately raises a fundamental question: what actually is BPD? [1,9,12]. Contributions from the last half century trying to answer this question have continuously uncovered a multiplicity of causes and long-term outcomes associated with BPD, gradually positioning “BPD” as an umbrella term, likely encompassing various endotypes and phenotypes, thus suggesting that its correct diagnosis and management belong to the realm of precision medicine.

BPD was first described by Northway and colleagues in 1967 as a lung injury in preterm infants resulting from oxygen and mechanical ventilation [13]. However, infants who develop BPD are typically more immature, often born at less than 28 weeks gestation, reflecting the changing epidemiology in the surfactant era. Advances in neonatal care and evolving respiratory support strategies have reshaped both the clinical course and the definition of BPD [14,15].

This narrative review examines how the evolving definitions reflect our expanding understanding of BPD pathogenesis, and evaluates the strengths and limitations of current animal models in elucidating disease mechanisms and their translational relevance. We performed a broad literature search of Scopus and PubMed using combinations of the keywords “bronchopulmonary dysplasia”, “hyperoxia”, “premature infant”, “hyperoxia-induced lung injury”, “animal models”, and “translational research”, which allowed identification of relevant in vivo studies (both clinical and pre-clinical), which were screened, analyzed, and narratively summarised for this review.

## 2. From Clinical Criteria to Disease Complexity: Evolving Concepts of BPD Phenotypes and Endotypes

### 2.1. The Early Era of Time-Point Definitions: From 28 Days to 36 Weeks PMA

Northway and colleagues first described BPD in a cohort of relatively mature preterm infants of 32 weeks gestation or older who required high concentrations of oxygen and mechanical ventilation in the pre-surfactant era. They described the radiological and histopathological findings of lungs severely damaged by ventilation and oxygen exposure [13].

In 1979 the first consensus emerged to establish a definition of BPD in the National Heart, Lung and Blood Institute (NHLBI)-sponsored workshop. Previously, Northway and colleagues had described the disease to establish criteria to define it, since its reports had high variability among neonatal units. Bancalari and colleagues established criteria based on preterm infants with hyaline membrane disease who had received mechanical ventilation. These criteria included persistent respiratory signs at 28 days of life, an oxygen requirement at 28 days to maintain a paO_2_ of at least 50 mmHg, and compatible radiologic abnormalities [16]. Shennan et al. introduced a pivotal shift in BPD classification by showing that the need for supplemental oxygen at 36 weeks PMA was more closely associated with later pulmonary morbidity than oxygen dependence at 28 days of life. This observation questioned the usefulness of the traditional 28-day criterion, particularly in very immature infants, and helped establish 36 weeks’ PMA as a more clinically meaningful time point for defining chronic lung disease of prematurity [17].

### 2.2. Defining Severity: The 2001 NHLBI Consensus

The definition proposed by the 2001 NHLBI consensus represented a shift in the conceptualization of BPD, as it introduced a classification of mild, moderate, and severe. This definition relied on oxygen requirement for at least 28 days (commonly operationalized as being on supplemental O_2_ at day 28 of life) and then assigned severity at 36 weeks PMA based on ongoing O_2_ and need for positive pressure; nevertheless, clinical application remains highly variable, with some institutions calculating cumulative days of exposure while others assess point-prevalence on day 28, prompting later definitions to abandon this metric [18]. A key contribution was the recognition that BPD is not a homogeneous disease, but rather a clinical spectrum whose severity depends on the persistence of the need for oxygen and/or positive pressure support. It better integrated the reality of the “new BPD” of the post-surfactant era, characterized by more immature newborns with less fibrosis and greater alteration of alveolar and vascular development [19].

Although these definitions have been clinically useful and widely adopted, they are based on variables that depend on the treatment decisions: the need for supplemental oxygen and/or positive pressure support. Their influence remains considerable, to the point of continuing to shape contemporary definitions of BPD. As a result, BPD classification may vary according to centre-specific respiratory management practice. A significant limitation, however, is their retrospective and relatively late timing, because they classify disease at 36 weeks PMA. In an extremely vulnerable population, early identification of disease trajectories may be crucial for prevention, risk stratification, and the development of targeted interventions.

### 2.3. Contemporary Challenges: Respiratory Support, Heterogeneity and Prediction

As the underlying physiology has become better understood, BPD has increasingly been recognized as a disorder of abnormal lung development, influenced by immaturity, inflammation injury, aberrant repair, and both prenatal and postnatal factors. In addition, changes in respiratory support practice, including the use of high-flow nasal cannula with room air or low-flow with 100% oxygen, have created clinical scenarios that are not adequately captured by earlier definitions. In 2016, a workshop at NICHD proposed reintroducing radiographic criteria, incorporating infants who died before 36 weeks PMA, and accounting for newer forms of noninvasive respiratory support such as high-flow nasal cannula [20].

In 2017, the Effective Perinatal Intensive Care in Europe (EPICE) cohort (22 + 0–31 + 6 weeks gestation across 11 countries) defined BPD as oxygen dependency or respiratory support at 36 weeks PMA. Subsequently, the Canadian Neonatal Network (CNN) showed that in infants <29 weeks gestation, evaluating support at 40 weeks PMA, rather than 36 weeks PMA, significantly improved the prediction of severe respiratory morbidity at 18–21 months’ corrected age [21].

In 2019, Jensen and colleagues proposed a BPD definition with improved prediction of morbidity by classifying disease severity according to the mode of respiratory support administered at 36 weeks PMA, regardless of the supplemental oxygen use. They proposed a three-grade classification based on the type of respiratory support at 36 weeks PMA: low-flow nasal cannula, noninvasive support, or invasive mechanical ventilation. It is among the most commonly used contemporary definitions [22]. Although the Jensen definition incorporates contemporary modes of respiratory support and improves the feasibility of BPD classification, several limitations remain: (i) severity is determined by the respiratory support prescribed at a single time point, (ii) it renders classification susceptible to institutional practice variation and (iii) it fails to capture evolving respiratory trajectories. Moreover, excluding oxygen concentration may overlook meaningful differences in gas-exchange impairment, while fixed nasal-flow thresholds may not provide comparable levels of respiratory support across infants [22,23] (Figure 1).

### 2.4. From the Definition to the Clinical and Biological Heterogeneity of BPD

BPD is a complex lung disease with diverse clinical presentations, variable pathogenic mechanisms, and a dynamic course over time. Operational definitions of BPD have enabled significant progress in identifying infants with severe disease during neonatal hospitalization, contributing to improved survival before discharge. However, current definitions do not fully capture the biological heterogeneity of BPD.

Phenotype-based descriptions of BPD have emerged in response to the limitations of traditional definitions based exclusively on oxygen use or respiratory-support requirements. Katz et al. conducted an international Delphi study to identify the key elements considered essential for developing a harmonised definition of BPD. The study included professionals involved in neonatal care and BPD research and highlighted substantial variation in the definitions used worldwide. The 2001 NIH definition was the most commonly used, followed by the 2019 Jensen definition and the 1988 Shennan definition. The authors reported that BPD definitions are primarily used for three purposes: quality improvement, prediction of long-term respiratory outcomes, and as outcome measures in research studies. Participants agreed that an optimal definition of BPD should enable meaningful comparisons between centres, reflect the quality of respiratory care, predict long-term respiratory and neurodevelopmental outcomes, and rely on clinical parameters that are readily accessible at the bedside. Importantly, there was strong consensus that BPD should be classified according to severity rather than treated as a binary diagnosis indicating the presence or absence of disease. This severity-based approach may allow for more individualised and clinically meaningful risk stratification. The study also emphasised that BPD should be understood as a heterogeneous condition, encompassing distinct clinical phenotypes that should be incorporated into future definitions [24].

Wu et al. described three major phenotype components in infants with severe BPD: pulmonary parenchymal disease, pulmonary hypertension and central airway disease; moreover, they observed the frequent coexistence of these components and that pulmonary hypertension and central airway disease were associated with the worst clinical outcomes [25].

Shepherd et al. demonstrated that infants with severe BPD exhibit different physiological phenotypes when assessed using infant pulmonary function testing, including obstructive, restrictive, and mixed patterns, with the obstructive phenotype being the most frequent. These findings support the concept that severe BPD encompasses heterogeneous pathophysiological pathways and suggest that functional characterization could improve patient stratification in future prognostic studies and therapeutic trials [26].

Laughon and colleagues pioneered approaches based on longitudinal clinical data trajectories and multi-phenotype construction to allow earlier recognition of disease patterns and more timely improvement of therapeutic strategies [27]. In this context, Alvarez-Paggi and colleagues [28,29] performed an exploratory analysis based on data from two independent prospective cohorts of preterm infants with a birth weight <1250 g and identified four distinct FiO_2_ requirements trajectories during the first 30 days of life.

Based on longitudinal FiO_2_ requirement trajectories, the authors identified four respiratory clusters: persistently low FiO_2_ requirement (PLR, 58%), initially high requirement with subsequent improvement (EHRI, 16%), late-onset high requirement (LOHR, 8%), and persistently high requirement (PHR, 18%). Mortality differed across groups, with higher rates in the LOHR and PHR groups (19% and 15%, respectively), while no deaths were observed in the PLR group and only one death in the EHRI group. Furthermore, 53% of deaths occurred before 36 weeks PMA, precluding a conventional diagnosis of BPD. These findings demonstrate that current definitions, based on oxygen or respiratory support at a fixed point in time, do not adequately capture the dynamic evolution of lung disease in prematurity. In contrast, trajectory-based approaches may allow for the early identification of high-risk subgroups, potentially better reflect clinical heterogeneity, and could support more precise and individualized interventions.

### 2.5. Towards the Discovery of BPD Endotypes

While clinical phenotypes describe the observable characteristics of a disease, the concept of endotypes focuses on the distinct underlying pathophysiological and molecular mechanisms that drive those clinical presentations [30]. This conceptual framework was first pioneered and successfully applied in other complex respiratory conditions, most notably asthma and bronchiolitis. In asthma, the identification of specific inflammatory endotypes (such as Type-2 versus non-Type-2 inflammation) revolutionized targeted biologic therapies, moving the field away from a one-size-fits-all approach [31]. Similarly, in severe viral bronchiolitis, distinct endotypes based on specific immune responses, microbiome profiles, and viral etiologies have been postulated to better predict severity and the subsequent risk of recurrent wheezing [32,33]. Applying this framework to respiratory medicine has underscored the importance of introducing mechanism-based characterizations to complement the already useful symptom-based categorizations.

In the context of BPD, adopting the concept of endotypes is particularly critical given the extreme vulnerability of the developing lung. BPD is not merely a disease of the neonatal intensive care unit; it is a condition that profoundly shapes long-term respiratory health [34]. The lung injury sustained can sensitize the infant’s respiratory tract, significantly increasing their susceptibility to severe viral bronchiolitis and ultimately elevating the risk of developing childhood and adult asthma [35]. Because the neonatal period represents a critical window of lung development, the prospective identification of specific BPD endotypes at this stage could potentially assist in anticipating long-term outcomes. Understanding the unique molecular and cellular pathways, such as specific inflammatory cascades, varying degrees of arrested alveolarization, or dysregulated angiogenesis, could enable clinicians to predict long-term sequelae and could ultimately inform earlier, more individualized interventions aimed at reducing future respiratory morbidity.

A recent synthesis formalizes BPD into three clinically actionable endotypes, namely parenchymal, airway, and vascular, and maps each to specific diagnostic modalities and potential targeted therapies [36] (Figure 2). The authors show that combining advanced lung imaging, infant pulmonary function testing, echocardiography, and biomarker panels improves endotype discrimination and prognostic stratification, and they advocate for AI-enabled phenotyping to guide endotype-stratified trials. Incorporating this framework strengthens the rationale for moving beyond oxygen-based severity scales toward precision enrollment and therapy selection in future studies. Concurrently, proposals to restructure the existing framework support the concept of prematurity-associated lung disease (PLD), aimed at improving the understanding and clinical management of preterm infants predisposed to chronic respiratory morbidity [34].

Transitioning toward endotyping in BPD requires, as an essential first step, the robust identification of clinical sub-groups or clusters that reflect distinct disease behaviors over time. In this regard, the trajectory-based approach described by Alvarez-Paggi and colleagues represents a significant translational milestone [28]. By identifying four distinct longitudinal clusters based on dynamic oxygen requirements, this approach moves beyond static definitions to capture the evolving clinical reality of the disease. These distinct clinical trajectories likely serve as macroscopic readouts of different underlying pathophysiological endotypes. Recognizing these dynamic respiratory clusters provides a foundational bridge: it allows researchers to systematically investigate the specific biomarkers and biological signatures associated with each trajectory, ultimately paving the way for the discovery of true BPD endotypes and the subsequent development of mechanism-specific therapies.

### 2.6. Bedside Identification of BPD Endotypes: Practical Approaches and Current Limitations

At present, neonatologists lack a single validated bedside test to assign patients to a specific BPD endotype; which is not unexpected considering that the endotypes themselves are currently being defined. A pragmatic clinical approach requires longitudinal, multimodal characterization that integrates daily respiratory trajectories with targeted structural and functional assessments. Different authors have proposed the existence of clearly defined endotypes, which are described below. Considering that the endotype framework applied to BPD is in its infancy, this list is expected to change depending on the ongoing research, the application of emerging technologies (see below, Section 4.4) and the validation of biomarkers.

Parenchymal Endotype: Clinical indicators such as persistent tachypnea, dyspnea, high supplemental oxygen requirement, hypercapnia, and growth faltering can be combined with structural imaging. Frameworks using chest radiography, computed tomography (CT; e.g., the Ochiai scoring system) [37], or magnetic resonance imaging (MRI) aid in identifying parenchymal structural impairment [38]. However, because CT and MRI are typically performed near term-equivalent age or discharge, require patient transport, sedation, or ionizing radiation exposure, their utility for routine, serial bedside monitoring in early life remains restricted.

Vascular Endotype: Identification relies primarily on serial echocardiography. Screening beginning at 36 weeks PMA and repeated until discharge yields high negative predictive value for BPD-associated pulmonary hypertension (BPD-PH) [39]. Notably, earlier screening (<32 weeks PMA) may identify a highly vulnerable subgroup, as approximately 40% of infants diagnosed with early BPD-PH die prior to discharge. Furthermore, pulmonary vascular abnormalities detected on echocardiogram as early as postnatal day 7 are associated with subsequent severe BPD and PH, though early predictive performance varies across cohorts [40].

Airway and Inflammatory Endotypes: Definitive identification of an airway endotype generally requires dynamic imaging or flexible bronchoscopy, precluding simple bedside diagnosis. Meanwhile, inflammatory biomarkers, while pathophysiologically informative, remain investigational and currently lack validated clinical thresholds for routine endotype assignment [36].

As clinical efforts shift toward discovering early bedside biomarkers for prompt endotypic characterization, animal models offer an essential translational tool to identify these early biological indicators and dissect their underlying pathophysiology.

## 3. Animal Models in BPD: Strengths, Weaknesses and Endotype-Based Classification

Animal models are important for investigating the mechanism of lung injury and repair in BPD and for evaluating potential therapies. Accordingly, animal models reproduce specific aspects of lung maturation, injury, and repair to help identify the mechanisms that promote either recovery or persistent lung disease [41].

The main strength of animal models is their capacity to isolate and study specific injurious mechanisms, including oxygen toxicity, mechanical ventilation and inflammation, across species at different stages of lung development [42]. Small animals, particularly rodents, offer several advantages: being born at the saccular stage of lung development, short life span, relatively low cost, reproducibility, and the possibility to manipulate genomic and molecular pathways. On the other hand, large animals such as lambs, baboons and piglets born in the alveolar stage more closely replicate human-like physiology and chronic respiratory conditions [43,44,45].

A major limitation of animal models is the lack of method and approach standardization, particularly in large animals, which may compromise clinical translation. Moreover, small animal species are born at the saccular stage of lung development, before completion of alveolarization; but, unlike preterm human infants, they can adapt to extrauterine life without requiring supplemental oxygen [1]. In addition, other clinically relevant modifiers such as chorioamnionitis, fetal growth restriction, or eventually fetal anomalies are also difficult to reproduce consistently. Finally, the high cost and sophisticated infrastructure requirements are major barriers to their widespread use.

In 1975 Northway and colleagues reported differential responses to three-day exposure to 100% oxygen in adult and newborn rodents. While the newborn rodents could survive but developed lung abnormalities similar to BPD, the adult rodents died rapidly [46]. Thus, model selection must account for the stage of lung development in the species under study. While the preterm human (24 to 36 weeks PMA) is born in a canalicular or saccular stage, rodents are born in the saccular stage at term. In contrast, rabbits, lambs, baboons, and humans born at term have lungs that are already in the alveolar stage [43,44,45].

The classification of animal models based on endotypes (Figure 2) could provide a useful framework for organizing preclinical research in a more specific and biologically relevant way. As extensively described in Section 2, BPD is increasingly recognized as a heterogeneous disease, rather than a single, uniform entity, with different injury patterns affecting the lung parenchyma, pulmonary vasculature, or neutrophilic/inflammatory pathways. In this context, animal models cannot be expected to reproduce the entire clinical spectrum of BPD. Their value lies, instead, in the extent to which they represent a specific component of the disease, a developmental window, or a pathogenic mechanism. Thus, animal models could be classified according to the endotype they represent the best by (at least partially) replicating the pathophysiological bases of a certain sub-phenotype, aiding researchers to select the most appropriate experimental approach to address specific research questions. For example, hyperoxia-based models can be particularly informative for studying impaired alveolarization and abnormal vascular development, while inflammation-based models may be better suited for exploring immune-mediated alterations in lung morphogenesis, extracellular matrix remodeling, or airway injury. Similarly, models incorporating mechanical ventilation or combined prenatal and postnatal insults may be more relevant when the goal is to study the complex interactions between lung immaturity and postnatal injury.

Therefore, classifying animal models by BPD endotype may support a more refined and targeted research strategy, without implying that a single model can fully capture the complexity of BPD. See Section 4.1 for endotype–animal model matching (Table 1).

### 3.1. Rodent Models: Hyperoxia, Genetic Manipulation, and Translation

Based on their birth at saccular stage [46], the neonatal rodent hyperoxia model typically involves exposing newborn pups to supplemental oxygen at different concentrations.

Namba et al. showed that neonatal exposure of mice to 95% oxygen for 96 h induces sex-specific long-term pulmonary sequelae. In adult males, neonatal oxygen exposure mainly affected pulmonary mechanics, increasing respiratory resistance and reducing the response to methacholine, without evident alveolar simplification. In contrast, adult females showed delayed or impaired alveolarization, while pulmonary mechanics remained relatively preserved. These findings suggest that the long-term consequences of neonatal hyperoxic injury may differ by sex, affecting airway function predominantly in males and alveolar structure predominantly in females [47,58].

Prolonged exposure to oxygen concentrations lower than 100% (65–85%) can cause irreversible lung injury, resulting in lesions closely resembling human BPD. This suggests that aberrant cell proliferation may play a critical role in the pathogenesis of chronic neonatal lung disease [59,60].

Rodent models demonstrate high experimental efficiency due to logistical advantages and low costs, factors that facilitate the management of large cohorts to ensure robust statistical analysis [61]. Their short gestation cycle of approximately 21 days significantly accelerates data generation compared to larger species. Furthermore, the larger size of rats relative to mice provides a technical advantage for surgical procedures and tissue collection. Similarly, the ability to derive in vitro models (2D or 3D) from these animals allows for high-throughput preliminary drug screening, aligning with the ethical mandate to reduce the number of subjects in later experimental phases [62,63].

Mice are widely regarded as a particularly valuable model in molecular research due to their unparalleled susceptibility to genomic manipulation and the sophistication of available biotechnological tools. Through the development of knockout models, specific genes can be silenced to elucidate precise molecular pathways; for instance, deleting the IL-6 and MMP-9 genes has been shown to reduce the severity of lung damage and protect against hyperoxia, respectively. Ultimately, this genetic analysis capability allows for the validation of critical biological functions and pathological mechanisms with a level of precision difficult to achieve in other models [64,65].

Furthermore, rodent models are useful for evaluating pharmacological interventions and elucidating complex molecular mechanisms. Recently, results obtained from these models have identified the tryptophan metabolite 3-hydroxyanthranilic acid (3-HAA) as a critical factor in the pathogenesis and treatment of BPD. Administering 3-HAA via nebulization (at an optimal dose of 75 mg/kg) significantly improved lung development in BPD rats. This was evidenced by an increased radial alveolar count (RAC), a decreased mean linear intercept (MLI), and a reduction in pro-inflammatory cytokines like TNF-α and IL-6 [66].

### 3.2. Large-Animal Models in BPD Research: The Translational Value of the Preterm Pig

Among large-animal models, pigs share important similarities with humans in body size, anatomical organization, immunology, genome structure, and physiology, making them particularly valuable for translational and clinical research applications when compared with rodents. Several additional features support their use as experimental models, including relatively short life cycles, large litter sizes, and the feasibility of genetic manipulation [67,68].

The anatomical and physiological similarities between pigs and humans provide a strong rationale for their use as biomedical models. Their comparable organ size and physiological responses, for example, allow the evaluation of safe dosage ranges in drug-development studies and pharmacological interventions. In the respiratory system, pigs and humans share similar pulmonary lobar and bronchial anatomy, which makes pigs useful for modelling respiratory diseases and testing therapeutic strategies. However, an important anatomical difference should be noted: humans have three lobes in the right lung and two in the left, whereas pigs have four lobes on the right and two on the left [55,67,69,70].

Neonatal pigs can undergo orotracheal intubation or direct tracheal cannulation and can be ventilated using conventional neonatal ventilators. These devices are commonly pressure-limited or pressure-controlled, meaning that inspiratory pressure is set while the delivered tidal volume may vary according to lung compliance and airway resistance. This feature allows neonatal pigs to be managed using respiratory-support strategies that closely resemble those applied in neonatal intensive care, strengthening their value as a large-animal model for translational respiratory research [71,72,73,74].

Additional advantages of the pig model include relatively low reproductive costs compared with other large animals, compatibility with genetic modification, and the ability to examine histological changes in the lung parenchyma in response to injury. These features make the pig particularly useful for studies aimed at linking physiological responses, ventilatory strategies, and structural lung injury [55,75]. For example, Frank Caminita et al. developed a preterm neonatal BPD model using preterm piglets that spontaneously developed respiratory distress syndrome. Mortality in non-ventilated animals was inversely related to gestational age and was attributed to respiratory failure with impaired gas exchange and immature alveolar architecture [76].

### 3.3. Ventilator-Injured Animal Models

Since the first description of BPD in 1967 involving newborns exposed to high concentrations of oxygen and invasive mechanical ventilation as described in Section 2, it was proposed that both factors played a role in the development of chronic neonatal lung injury [13]. In this context, the first animal models focused primarily on exposure to hyperoxia, with the aim of reproducing oxygen-induced lung damage and analysing its underlying biological mechanisms [46,77].

With advances in neonatal mechanical ventilation, alongside the improved survival of extremely preterm infants, BPD gradually became a different clinical entity [14]. This transition created a need to develop animal models that incorporated not only hyperoxia, but also mechanical ventilation and the context of preterm birth, in order to better understand the pathophysiology of the disease. In this regard, studies in premature rabbits explored the physiological effects of surfactant in animals exposed to hyperoxia and subjected to mechanical ventilation, with or without surfactant administration [42,78]. These models demonstrated that mechanical ventilation increased the transfer of proteins from the circulation into the alveolar interstitium, a phenomenon that could be prevented by surfactant administration and was associated with alveolar damage on histological analysis [79].

Models in premature baboons provided a closer approximation to the clinical scenario of BPD. A study of premature baboons with hyaline membrane disease confirmed the presence of BPD from a histopathological perspective, characterised by a heterogeneous pattern of pulmonary inflation, with areas of atelectasis and overdistension or ‘emphysematous’ changes, bronchial and bronchiolar lesions with necrosis, hyperplastic regenerative changes and/or squamous metaplasia, and peribronchiolar fibrosis and incipient alveolar wall fibrosis [56].

In both premature baboon and premature lamb models, preterm birth associated with prolonged mechanical ventilation reduces pulmonary capillary growth. In premature lambs, studies have shown that atrophic secondary alveolar septa lack capillaries, indicating impaired microvascular development. Furthermore, prolonged mechanical ventilation is associated with a reduction in the number of pre- and post-capillary arterioles and venules. Consequently, there is a reduction in the growth of the pulmonary microvascular bed, a phenomenon that contributes to the respiratory failure observed in these ventilated animal models [57,80].

The pulmonary histopathology of progressive neonatal chronic lung disease, as observed in baboon and preterm lamb models, closely mirrors the abnormalities described in premature infants with BPD [80,81]. In particular, models subjected to chronic ventilation show alveolar simplification, persistent muscularisation of the pulmonary arterioles and increased bronchiolar muscularisation. These characteristics reflect not only lung injury, but also a disruption of alveolar and vascular development, central aspects of contemporary BPD. Animal models in premature lambs allow for the longitudinal study of pulmonary changes during the course of the disease. Meanwhile, in the premature baboon model, it has been demonstrated that alterations in secondary alveolar septation, capillary growth and microvascular development may persist into later stages of life. These findings highlight the utility of large-animal models for investigating the long-term consequences of neonatal lung injury [80,81,82,83].

Furthermore, models in premature baboons and lambs have provided relevant information on respiratory mechanics and airway resistance. Studies show that prolonged mechanical ventilation is associated with an excessive increase in expiratory airway resistance and a decrease in lung compliance [84]. In addition, in premature lambs, prolonged mechanical ventilation induces an increase in the thickness of the smooth muscle layer around the bronchioles, suggesting small airway remodelling [85].

Taken together, large-animal models, including premature lambs, sheep and premature baboons, are fundamental tools for translational research into BPD, as they provide information that is difficult to obtain using small-animal models. Premature lamb and baboon models more faithfully replicate the clinical context of preterm birth, respiratory failure, the need for prolonged ventilatory support, and exposure to oxygen therapy for days or weeks. A particular advantage of the premature lamb model is its larger size, which facilitates serial physiological studies throughout the course of the disease, continuous haemodynamic monitoring via permanent vascular catheters, the measurement of pulmonary and systemic parameters, as well as the frequent collection of blood samples and other biological fluids.

Other animal models, such as fetal sheep, particularly those utilizing in utero ventilation (IUV), serve as premier large-animal systems that closely replicate extremely preterm respiratory physiology. These models facilitate the isolation of ventilation-induced lung injury (VILI) and associated vascular alterations, such as increased pulmonary arteriolar wall thickness, without the confounding systemic interventions of ex utero life [86,87,88].

These advantages allow for the integration of morphological, biochemical and molecular analyses aimed at identifying the role of specific genes and pathways in the pathogenesis of BPD. Furthermore, studies in large-animal models have demonstrated that the mode of ventilatory support influences pulmonary outcomes. In particular, invasive mechanical ventilation is associated with greater injury and impaired lung development compared with less invasive strategies, such as high-frequency nasal ventilation. Thus, these models not only allow the study of the mechanisms of lung damage and repair, but also the evaluation of ventilatory and therapeutic strategies with potential clinical relevance for the prevention and treatment of BPD [89,90,91].

### 3.4. Inflammatory Models Across Species: LPS and Double-Hit Exposures

Rodents, particularly rats and mice, are widely used in experimental BPD research because key stages of lung development, including the saccular and alveolar stages, occur postnatally. This developmental timing facilitates experimental manipulation of the processes involved in alveolarisation and vascular growth [51,52,92]. In contrast, large-animal models, including piglets, sheep, and baboons, have been particularly valuable for studying the effects of mechanical ventilation, oxygen exposure, and chorioamnionitis in structurally and physiologically immature lungs [42,88,93,94].

One of the most informative approaches for modelling inflammation-associated BPD is the “double-hit” strategy, in which prenatal inflammation is combined with postnatal hyperoxic exposure. In these models, intra-amniotic administration of lipopolysaccharide (LPS) is commonly used to mimic chorioamnionitis. Prenatal inflammatory exposure appears to sensitise the immature lung to subsequent oxygen-induced injury, resulting in more pronounced impairment of alveolarisation and pulmonary vascular development [52].

Advances in genetic engineering have also enabled the development of mouse models with conditional activation or overexpression of specific inflammatory mediators. Conditional activation of the NF-κB pathway in the respiratory epithelium induces robust neutrophilic inflammation and has shown that the saccular stage represents a critical window of susceptibility. During this period, inflammation can disrupt elastin assembly through neutrophil elastase activity, leading to persistent airspace enlargement and increased susceptibility to chronic obstructive pulmonary disease-like changes in adulthood [51,53].

Similarly, perinatal overexpression of the cytokine IL-1β is sufficient to induce a BPD-like lung phenotype, characterised by alveolar hypoplasia, airway remodelling, and abnormal capillary development, even in the absence of hyperoxic exposure [54].

These models have also shown that epithelium-derived inflammation alters key extracellular matrix components required for distal lung development. In particular, downregulation of fibulin-5 and elastin, both essential for the structural integrity of terminal airspaces, has been observed. Experimental restoration of fibulin-5 has been shown to rescue elastic fibre assembly, suggesting that extracellular matrix pathways may represent potential therapeutic targets [51,53].

Animal models have further provided a platform for testing anti-inflammatory interventions. For example, administration of interleukin-1 receptor antagonist (IL-1Ra) has been shown to attenuate lung structural disruption induced by perinatal inflammation and moderate hyperoxia, reducing the activation of macrophages and dendritic cells and preserving aspects of alveolar and vascular development [92].

Overall, animal models have helped establish that inflammation is not merely a secondary consequence of lung injury, but a central driver of BPD pathogenesis. Through mediators such as IL-1β and NF-κB, inflammatory signalling can interfere with the genetic and structural programmes that regulate early lung development, thereby contributing to impaired alveolarisation, abnormal vascular growth, and long-term respiratory vulnerability [95,96].

### 3.5. Matching the Model to the Research Question

In this context, the value of an animal model should not be assessed solely on the basis of whether it reproduces the full clinical spectrum of human BPD, particularly because, as has been extensively discussed above, the disease itself is highly heterogeneous. Thus, no single animal model can fully capture the complexity of the condition, which reflects the interaction of developmental immaturity, prenatal exposures, postnatal injuries, and clinical management practices. Therefore, the selection of an experimental model should be guided by the specific biological question being addressed.

An animal model may be particularly informative when it reflects the stage of lung development at the time of injury, the nature and timing of the injurious exposure, the cellular mechanism or molecular pathway under investigation, or the therapeutic mechanism being tested. For example, hyperoxia-based models may be useful for studying oxygen-mediated disruption of alveolar and vascular development, whereas inflammation-based or combined-injury models may be more appropriate for investigating prenatal or postnatal inflammatory pathways. Similarly, large-animal models may offer advantages when the research question requires a closer approximation to human lung structure, respiratory support strategies, or translational therapeutic testing.

Thus, when selecting an animal model, experimental design should prioritize the concordance between the model and the specific aspect of BPD pathobiology under investigation. This approach allows animal models to be interpreted as complementary tools that reproduce selected mechanisms or developmental vulnerabilities, rather than as complete replicas of the human disease.

## 4. Methodological Recommendations and Future Directions

To advance the translational impact of preclinical research in BPD, the scientific community may need the transition from treating BPD as a single, uniform entity toward an objective, endotype-driven approach. Based on the clinical and preclinical evidence reviewed in the preceding sections, several recommendations are outlined below.

### 4.1. Align Preclinical Models with Specific Clinical Endotypes

A single animal model cannot replicate the entire clinical syndrome of BPD, so researchers would benefit from platforms that accurately mimic specific pathological endotypes (as outlined in Figure 2 and further expanded in Table 1), such as the following:

- For investigations targeting the Neutrophilic/Inflammatory Endotype, multi-hit models combining prenatal chorioamnionitis (e.g., intra-amniotic LPS) with postnatal hyperoxia or mechanical ventilation are highly recommended [51,52,53,54].
- Studies focused on the Vascular Endotype (e.g., pulmonary hypertension, impaired capillary angiogenesis) should utilize prolonged hyperoxia in neonatal rodents or sustained mechanical ventilation in larger mammals, where vascular remodeling is a primary driver [47,48,49,50].
- For the Parenchymatous Endotype, characterized by alveolar simplification and architectural remodeling, neonatal rodent hyperoxia remains a robust, reproducible, and high-throughput tool [55,56,57] (Table 1).

### 4.2. Standardize Protocols and Implement Multimodal Longitudinal Readouts

To potentially improve reproducibility across laboratories, future studies could move away from arbitrary fixed-percentage hyperoxia protocols unless strictly justified. Preclinical designs would ideally report precise oxygen concentrations, transition timelines (abrupt vs. gradual return to room air), and precise tidal volumes or ventilation pressures in large-animal models to better match modern protective ventilation strategies used in Neonatal Intensive Care Units. Furthermore, biomarker discovery and therapeutic testing in animal models could encompass longitudinal assessments rather than single endpoints. Preclinical pipelines might advantageously combine structural histology (such as Mean Linear Intercept) with functional readouts (e.g., flexiVent respiratory mechanics, echocardiography for right ventricular hypertrophy) and deep molecular profiling (single-cell RNA sequencing or spatial transcriptomics) to better map the cellular trajectories of injury and repair [97,98,99].

### 4.3. Limitations of Current Translational Frameworks

While animal models have provided foundational insights into the pathophysiology of neonatal lung injury, several inherent limitations constrain their direct translation to human infants. First, the widespread use of term-born rodents exposed to hyperoxia bypasses the critical systemic complexities of human extreme prematurity, including an immature immune system, metabolic instability, and systemic organ cross-talk (e.g., the gut–lung or brain–lung axis). Conversely, while large-animal models (lambs, baboons, pigs) successfully incorporate preterm birth and intensive care management, their implementation is severely restricted by high financial costs, specialized infrastructure requirements, low throughput, and limited tools for genetic manipulation. Furthermore, current preclinical designs rarely account for sexual dimorphism or genetic background heterogeneity, both of which are prominent risk factors in human clinical cohorts.

### 4.4. Future Directions and Emerging Technologies

To bridge these translational gaps, the next generation of BPD research must integrate complex in vivo modeling with emerging in vitro and in silico technologies:

- Ex vivo precision-cut lung slices (PCLS) preserve the native three-dimensional multicellular architecture and cell–matrix interactions of the lung parenchyma. By isolating localized lung responses from systemic immune and circulatory influences, this flexible, high-throughput platform has successfully enabled the identification of localized molecular pathways, such as hyperoxia-triggered, NF-κB-dependent mesenchymal Wnt5a expression, while directly adhering to the ethical principles of the 3Rs (replacement, reduction, and refinement) [100].
- Human-Based Complex In Vitro Models: The optimization of lung-on-a-chip technologies and multi-lineage human induced pluripotent stem cell (iPSC)-derived lung organoids will allow researchers to model human-specific cellular responses to hypoxia, hyperoxia, and mechanical stretch under controlled microfluidic conditions [44,101].
- Multi-Omics and Systems Biology: Applying spatial transcriptomics, single-cell sequencing, and proteomics to both preclinical tissue and clinical biofluids (e.g., tracheal aspirates) will help identify shared molecular signatures, refining the accuracy of animal-to-human translation [102,103,104].
- Artificial Intelligence and Predictive Modeling: The integration of machine learning (ML) and deep learning (DL) into neonatal respiratory research is transforming risk stratification and endotype discovery. Recent breakthroughs span three complementary pillars: (1) AI-based imaging phenotyping (e.g., U-Net and CNN architectures) applied to early neonatal chest radiographs that enable automated lung segmentation and early prediction of BPD severity [105], (2) unsupervised clustering and data-driven trajectory modeling using unsupervised machine learning algorithms applied to longitudinal clinical data for unbiased, earlier risk stratification [28] and (3) physiological time-series and long-term functional forecasting using recurrent neural networks (RNN) trained on non-invasive tidal breathing data that can discriminate BPD severity in the NICU [106]. These approaches could help discover early biomarkers of specific endotypes, ultimately paving the way for personalized, preventive therapeutic interventions in high-risk preterm infants [28,105].

## 5. Concluding Remarks

BPD has evolved from an entity initially defined by oxygen toxicity and ventilator-associated lung injury to a heterogeneous developmental lung disease characteristic of extreme prematurity. Although successive definitions have improved clinical classification and the prediction of some outcomes, most continue to be based on the requirement for oxygen or respiratory support at fixed points in time. Consequently, these definitions fail to fully capture the dynamic trajectories, biological diversity, and long-term consequences of the disease. One of the main current challenges is to move beyond retrospective diagnosis based solely on clinical characteristics and intervention variables toward a framework that integrates clinical phenotypes, longitudinal respiratory trajectories, and endotypes defined by pathophysiological mechanisms.

This approach could allow for earlier risk stratification with the aim of improving patient selection for targeted interventions and strengthening the prediction of long-term respiratory and cardiopulmonary outcomes. In this context, animal models remain important tools, because they allow for the isolation and investigation of specific developmental windows, concrete injury exposures, and particular molecular pathways. Thus, in combination, they allow the exploration of the entire spectrum of human BPD.

Therefore, future translational research should prioritize the concordance between the experimental model and the biological question being addressed. Further research is needed to optimize injury protocols using multimodal, longitudinal assessments and integrating multi-omics platforms.

Ultimately, redefining BPD from an endotype-based and translation-oriented perspective could help bridge the gap between preclinical findings and individualized neonatal care. This shift towards a precision medicine-based approach may support the development of preventive and therapeutic strategies aimed not only at improving survival but also at reducing lifetime respiratory morbidity in extremely preterm infants.

## Figures and Tables

**Figure 1:**
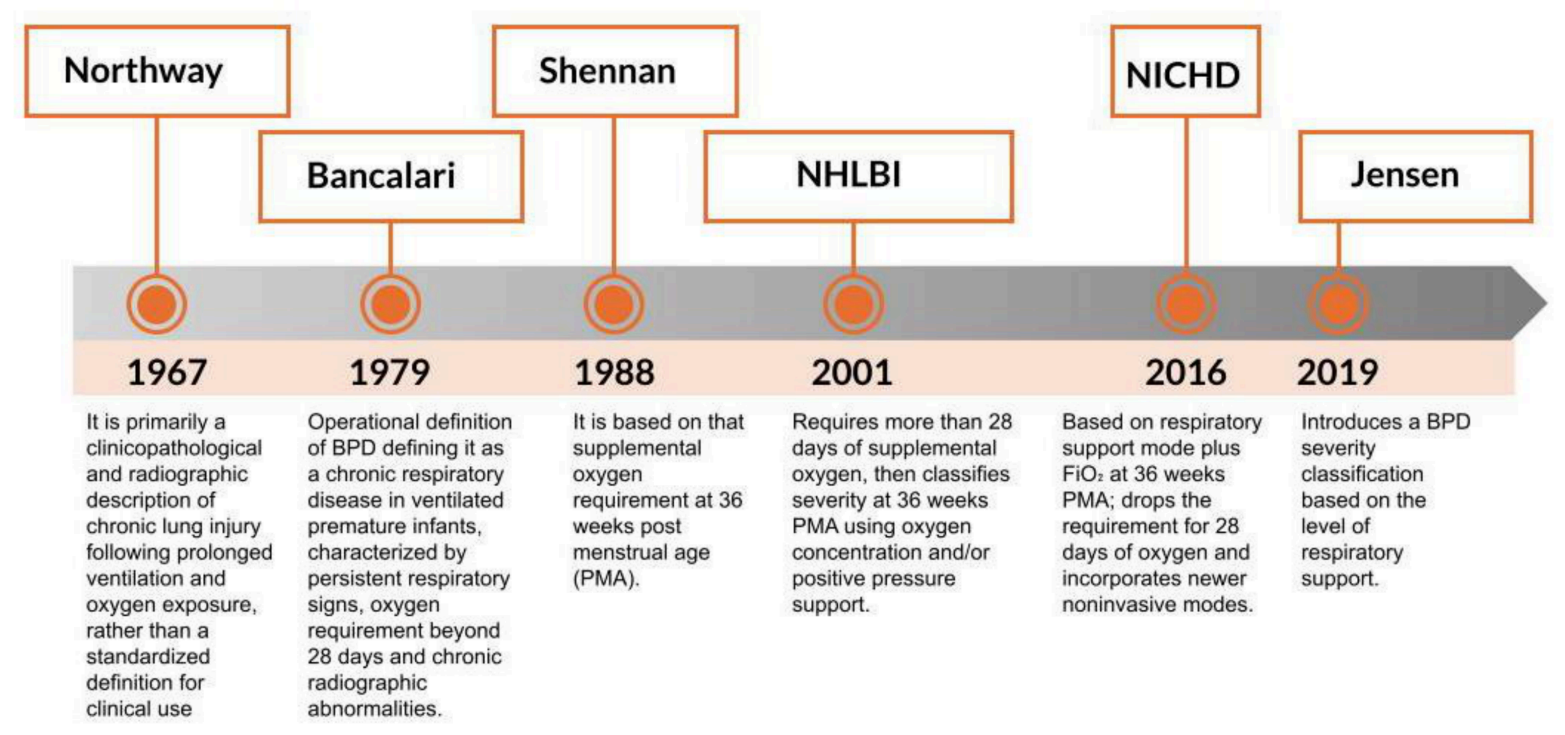
Evolution of BPD Definitions and Assessment Criteria. NIH: National Institute of Health, NHLBI: National Heart, Lung, and Blood Institute, NICHD: National Institute of Child Health and Human Development, PMA: postmenstrual age.

**Figure 2:**
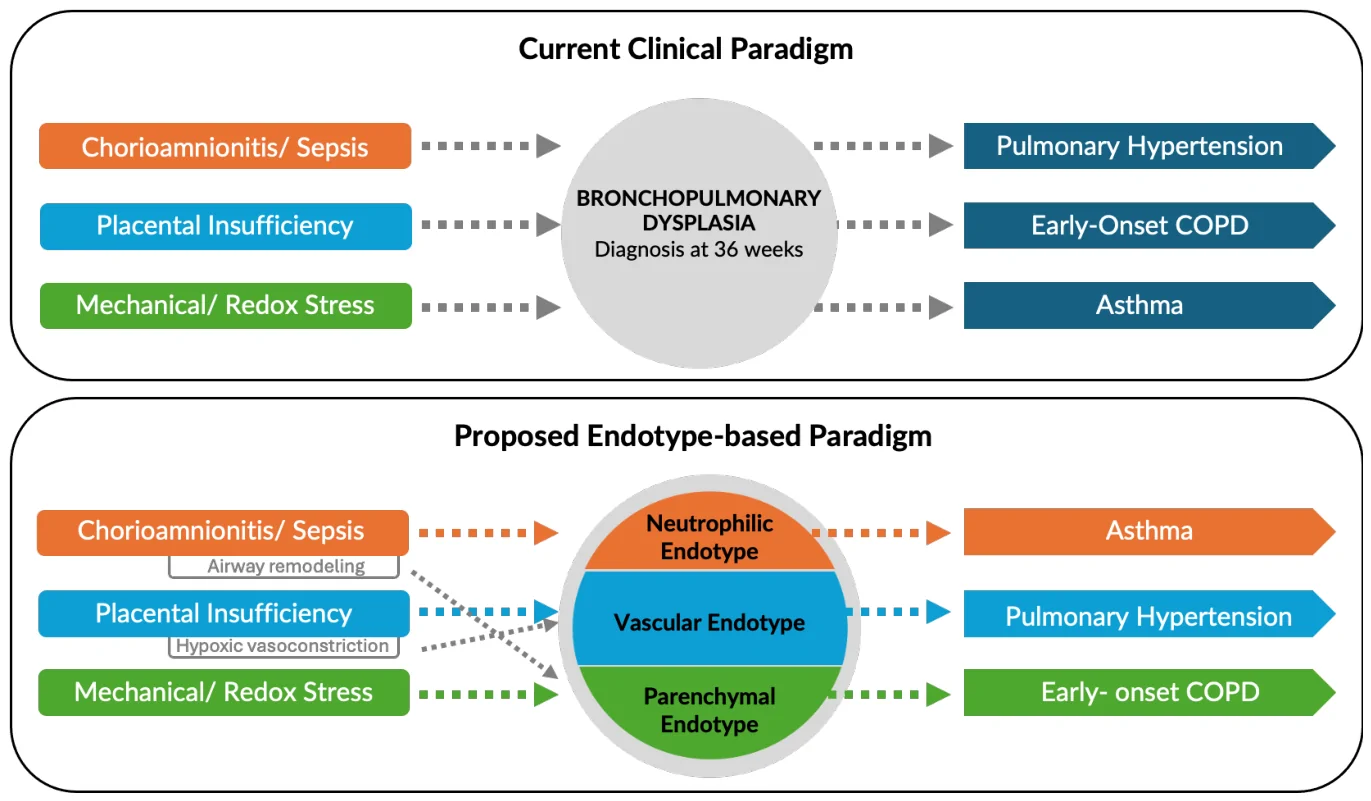
Shifting paradigms in BPD classification. (**Top**) The Current Clinical Paradigm: Diverse antenatal and postnatal insults during lung development converge into a single umbrella diagnosis based on oxygen requirement at 36 weeks PMA, obscuring underlying pathophysiology and confounding the prediction of long-term pediatric outcomes. (**Bottom**) The Proposed Endotype Paradigm: Disentangling BPD into distinct pathobiological endotypes (e.g., vascular, inflammatory, mechanical) preserves mechanistic continuity, allowing for targeted therapies in the NICU and precise prediction of specific long-term sequelae (e.g., pulmonary hypertension vs. early-onset COPD). Specific endotype-to-phenotype trajectories shown in the Bottom Panel are speculative and represent working hypotheses based on current translational evidence, requiring validation in prospective, precision-medicine cohorts.

**Table 1:** Endotype/phenotype- based classification of experimental animal models of BPD. Representative preclinical models of BPD are summarized according to the predominant endotype.

Species/Animal Model	Stage of Lung Development	Injury Protocol	Primary Mechanism/Endotype	Main Outcomes Assessed	Translational Relevance/Limitations	Ref.
**Neonatal mice or rats; preterm rabbits**	Saccular-to-early alveolar stage in neonatal rodents; gestational age-dependent late canalicular or saccular stage in preterm rabbits	Postnatal hyperoxia (85% O_2_ in rodents); hyperoxic exposure in preterm rabbits	**Vascular endotype:** impaired angiogenesis and VEGF/FLT1 signaling, distal vascular remodeling, and muscularization of small pulmonary arteries	Pulmonary vascular remodeling, angiogenic signaling, right ventricular hypertrophy, and echocardiographic indicators of pulmonary hypertension	Enables serial echocardiography and high-throughput molecular studies but lacks the mechanical volutrauma associated with NICU respiratory support.	[47,48,49,50]
**Rodents or sheep exposed to antenatal LPS; genetically modified rodents, including IL-6−/− and MMP-9−/− strains**	Fetal canalicular or saccular stage, depending on species and timing of exposure; neonatal rodents are born during the saccular stage	Antenatal LPS exposure or targeted disruption inflammatory pathways, alone or combined with postnatal injury	**Inflammatory/neutrophilic endotype:** chorioamnionitis-related pathways, increased IL-6, TNF-α and IL-1β signaling, and extracellular matrix remodeling	Cytokine expression, neutrophilic infiltration, extracellular remodeling, and impaired alveolar development	Useful for distinguishing prenatal from postnatal inflammatory mechanisms and evaluating targeted biologic or pharmacological interventions. Rodents, however, adapt to extrauterine life without requiring the respiratory stabilization commonly needed by very preterm infants.	[51,52,53,54]
**Preterm ventilated lambs or baboons; preterm piglets undergoing orotracheal intubation**	Late canalicular-to-saccular stage, depending on species and gestational age	Preterm delivery followed by invasive ventilation, with clinically relevant oxygen, ventilatory, and surfactant strategies	**Parenchymal/mechanical endotype:** volutrauma, mechanical shear stress, alveolar simplification, septal thickening, altered compliance, and fibrotic remodeling	Lung compliance, alveolar and septal morphology, pulmonary inflammation, fibrotic remodeling, and ventilator-induced lung injury	Reproduces key aspects of NICU care, including intubation, mechanical ventilation, and surfactant administration. Limitations include high infrastructure costs, small sample sizes, and limited opportunities for genetic manipulation.	[55,56,57]

VEGF/FLT1: Vascular Endothelial Growth Factor/Fms-like tyrosine kinase 1, LPS: Lipopolysaccharide, IL-6: interleukin-6, MMP-9: Matrix Metalloproteinase-9, Tumor Necrosis Factor-alpha.

## Data Availability

No new data were created or analyzed in this study. Data sharing is not applicable to this article.
